# Behavioral and Thermoregulatory Responses to Changes in Ambient Temperature and Wheel Running Availability in *Octodon degus*

**DOI:** 10.3389/fnint.2021.684988

**Published:** 2021-06-30

**Authors:** Beatriz Bano-Otalora, Maria Angeles Rol, Juan Antonio Madrid

**Affiliations:** ^1^Chronobiology Lab, Department of Physiology, Faculty of Biology, University of Murcia, Espinardo, Campus Mare Nostrum, IUIE, IMIB-Arrixaca, Murcia, Spain; ^2^Ciber Fragilidad y Envejecimiento Saludable (CIBERFES), Madrid, Spain

**Keywords:** *Octodon degus*, dual-phasing species, locomotor activity rhythm, body temperature rhythm, nocturnal, diurnal, masking, wheel running availability

## Abstract

*Octodon degus* is primarily a diurnal species, however, in laboratory conditions, it can switch from diurnal to nocturnal in response to wheel running availability. It has been proposed that this activity inversion obeys thermoregulatory constraints induced by vigorous physical exercise. Thus, its activity shifts to the night as the ambient temperature is lower.Here, we investigate the relationship between thermoregulation and the activity phase-inversion in response to wheel-running in this species. We measured behavioral activity and body temperature rhythms in diurnal naïve animals under 12 h light: 12 h dark cycles at four different ambient temperatures (spanning from ~26°C to 32°C), and following access to running wheels while maintained under high ambient temperature.Our results show that naïve degus do not shift their diurnal activity and body temperature rhythms to a nocturnal phase when subjected to sequential increases in ambient temperature. However, when they were provided with wheels under constant high-temperature conditions, all animals inverted their diurnal phase preference becoming nocturnal. Both, negative masking by light and entrainment to the dark phase appeared involved in the nocturnalism of these animals. Analysis of the thermoregulatory response to wheel running revealed some differences between masked and entrained nocturnal chronotypes.These data highlight the importance of the coupling between wheel running availability and ambient temperature in the nocturnalism of the degus. The results support the view that an innate “protective” pre-program mechanism (associating darkness and lower ambient temperature) may change the timing of behavioral activity in this species to reduce the potential risk of hyperthermia.

## Introduction

Temporal niche selection in mammals results from the integrated actions of the central pacemaker and other internal or external cues that adaptively sculpt the overt circadian rhythms, without affecting the phase of the brain central pacemaker (otherwise known as masking; Mrosovsky, 1999).

*Octodon degus* is a caviomorph rodent from Central Chile, which has been described as diurnal with major episodes of activity at dawn and dusk (Fulk, 1976). Under specific laboratory conditions, however, diurnal, nocturnal, and crepuscular chronotypes emerge in this species (Labyak et al., 1997; García-Allegue et al., 1999), offering a unique opportunity to investigate the mechanisms underlying temporal niche preference in mammals. Chronotype flexibility is observed in other mammals (Hut et al., 2012) including Nile grass rat (Blanchong et al., 1999), golden spiny mouse (Levy et al., 2007), Mongolian gerbils (Weinert et al., 2007), and tuco-tucos (Tachinardi et al., 2015). Some authors have shown that degus shift their activity and body temperature rhythms from diurnal to nocturnal pattern in response to unrestricted wheel running access (Kas and Edgar, 1998, 1999; Vivanco et al., 2009).

Under natural environment, expression of a specific chronotype may be important in degus, serving as an adaptive process to predator-prey relationship cycle, seasonal changes, or food availability (Lagos et al., 1995; Kenagy et al., 2002; Vivanco et al., 2010a), for example. In the laboratory, it has been shown that degus not only select the dark phase with cooler ambient temperature to run in wheels (Vivanco et al., 2010c, d) but prefer to be active during the subjective night even when the ambient temperature did not change throughout the circadian day (Kas and Edgar, 1999). This raises the possibility that thermoregulation may be an endogenous overriding factor in determining nocturnal behavioral activity in this species.

Thus, chronotype flexibility in degus could represent a form of adaptive behavioral thermoregulation, which prevents animals from overheating during the day, confining their activity to the night when it is cooler, or when is associated with a cooler temperature to be active. This agrees with observations in the wild, reporting an increase in the degus’ diurnal activity during winter days, and a limitation of its activity to dawn and dusk during the hot and dry days of summer (Kenagy et al., 2002).

To better understand the relationship between thermoregulation and phase-switching responses of degus to wheel-exposure, we measured behavioral activity and body temperature rhythms in diurnal naïve animals under 12 h light: 12 h dark cycles at four different ambient temperatures spanning from 26°C to 32°C, which is in the upper limit of the degus’ thermoneutral zone (Rosenmann, 1977). Second, while degus were exposed to high ambient temperature, running wheels were provided. Previous studies show that some nocturnal degus are entrained to the night phase, while others become nocturnal by negative masking when they have free access to running wheels (Vivanco et al., 2009; Otalora et al., 2013). Here, we also assessed if entrained and masked nocturnal degus have fundamental differences in their thermoregulatory response.

## Materials and Methods

### Animals and Housing Conditions

Male degus (*n* = 16, 18–20 months old) with no wheel running experience (naïve) were obtained from a colony established at the Animal Service at the University of Alicante (Spain). Animals were maintained grouped housed under 12 h light: 12 h dark (L:D) cycle until taken for the study.

Animals were then individually housed in plexiglas cages kept under LD 12:12 cycle in a cabinet with controlled temperature and relative humidity (60% ± 10%). Zeitgeber Time (ZT) 0 corresponds to the time of lights on, and ZT 12 to lights off. Light was provided by two lateral fluorescent lamps controlled by an electronic timer (Data Micro, Orbis, Madrid, Spain), with an intensity between 350–400 lux at cage level. At a specific part of the experiment (stage 5; see below), running wheels were introduced for the first time (52 × 15 × 27 cm, length × height × width) while degus were exposed to high ambient temperature. Degus were fed with commercial rat chow (A04-rat-mouse maintenance Panlab, Barcelona, Spain) and drinking water *ad libitum*. Ambient temperature was recorded every hour by a data logger iButton placed on a shelf in the middle of the room.

All experimental procedures were performed in accordance with the guidelines issued by the Spanish Ministry of Agriculture, Fishing, and Feeding (Royal Decree 1201/2005 of October 21, 2005).

### Data Recording

Body temperature (Tb) was recorded every 60 min using a datalogger (ThermoChron^®^, Data loggers iButton; Maxim Integrated Products, Sunnyvale, California, United States), with an accuracy of 0.125°C. Data loggers were implanted intraperitoneally under aseptic conditions, as previously described (Vivanco et al., 2007). Isofluorane was used as an anesthetic (Forane^®^; Abbot Laboratories S.A., Madrid, Spain) and iodine solution (Betadine^®^; Viatris, Madrid, Spain) as a surgical antiseptic. Absorbable sutures (2–0; Safil^®^ Quick; B/Braun, Barcelona, Spain) were used to suture the abdominal muscle layer, and non-absorbable silk (2–0; Silkam^®^; B/Braun, Barcelona, Spain) was employed to suture the skin. No mortality or morbidity was observed after the surgery. Animals were allowed to recover for a week. Data collected during the recovery period were excluded from data analysis. In the middle of stage 4 (see below), new surgeries were performed to replace the data loggers. Once data loggers were removed, data were transferred to a computer. Body temperature recordings from a total of 10 animals were used for analysis.

During the period without wheels, locomotor activity was recorded in 10 min bins using infrared motion sensors (OMROM E3S-AD62; OMROM Corporation, Kyoto, Japan). Data obtained were normalized between 0 and 1, considering data above 95% percentile as 1. Activity sensors were installed on the long side of each plastic cage and were connected to a data acquisition system (Electronic Service at the University of Murcia, Murcia, Spain). With such a system, an output signal is generated each time the animal interrupts the infrared light beam.

Wheel running activity (WRA) was recorded as wheel revolutions/10 min intervals using the same acquisition system as for locomotor activity.

### Experimental Design

Degus with no prior wheel running experience (naïve) were subjected to four stages with sequential increments in ambient temperature during 104 days defined as follows (values are expressed as mean ± SD): Stage 1 (20 days, 26.0 ± 0.8°C); Stage 2 (13 days, 28.3 ± 0.6°C); Stage 3 (21 days, 29.8 ± 0.8°C) and Stage 4 (50 days, 32.5 ± 0.7°C). Then, running wheels were provided, and animals were allowed to run for another 21 days (Stage 5 (33.2 ± 0.8°C) divided into: Stage 5.1 corresponding to the first 10 days and stage 5.2 corresponding to days 11–21) before room temperature was reverted to 30°C for the next 10 days (Stage 6, 30.5 ± 0.7°C). After it, animals were transferred for 5 days into constant darkness (DD), and then finally returned for 10 more days to their original 12:12 LD cycle.

### Chronotype Characterization and Data Analysis

Degus chronotype was determined based on the percentage of daily activity exhibited during photophase. Degus with <40% of activity during the day were considered nocturnal. As some nocturnal degus are entrained to the dark phase while others become nocturnal by negative masking (Vivanco et al., 2009), we assessed negative masking by transferring nocturnal degus from LD conditions to constant darkness (DD) and then assessed their phase during the first few days in DD. Nocturnal degus who started to free run from their former LD phase when placed under DD were considered “entrained nocturnal” (EN); whereas those which showed an immediate phase advance (between 2–6 h) from the first days under DD so that they ran in their subjective day were defined as “masked nocturnal” (MN). Assessments were based on visual inspection of each actogram by three experienced researchers.

Locomotor activity, WRA, and Tb actograms were generated using El Temps© (version 1.228: A. Díez-Noguera, University of Barcelona). Averaged mean waveforms and quantitative analysis of Tb and activity rhythms were performed for a period of 8 days from each experimental stage. To avoid interference with masking effects induced by light–dark transitions, data from 60 min before and 60 min after light–dark transitions were excluded from the quantitative analysis. Inter-daily stability (constancy of the 24-h rhythmic pattern over days); intra-daily variability (rhythm fragmentation), and relative amplitude (based on the most active 10 h period and the least active 5 h period), were also calculated (Van Someren et al., 1999; Lax et al., 2011).

ANOVA with repeated measures was performed to assess differences in the activity and Tb rhythm parameters across different experimental stages. Two-way ANOVA was used to compare differences in WRA and Tb between the time of day and experimental stages. Significant interactions were followed by pairwise comparisons using paired Students *t*-test with Bonferroni correction. Paired or unpaired Student’s *t*-test was performed when only two groups were compared (e.g., time of the day (light phase vs. dark phase) or chronotypes (“EN vs. MN”). Values of *p* < 0.05 were considered to be statistically significant.

Although we tried our best to maintain ambient temperature as constant as possible between days within each stage, there was some variation. Thus, a linear regression analysis was performed to assess the relationship between activity and body temperature with ambient temperature using data for each particular day across stages with or without wheels. *p* < 0.05 indicates that the slope of the fitted line is significantly non-zero.

Data are expressed as mean ± SEM, unless otherwise stated. Specific sample sizes can be found in the figure legends and results section. All statistical tests were performed using SPSS version 23 (SPSS Inc., Chicago, IL, USA) and GraphPad Prism 7.04 (GraphPad Software Inc., San Diego, CA, USA).

## Results

**Figure 1:**
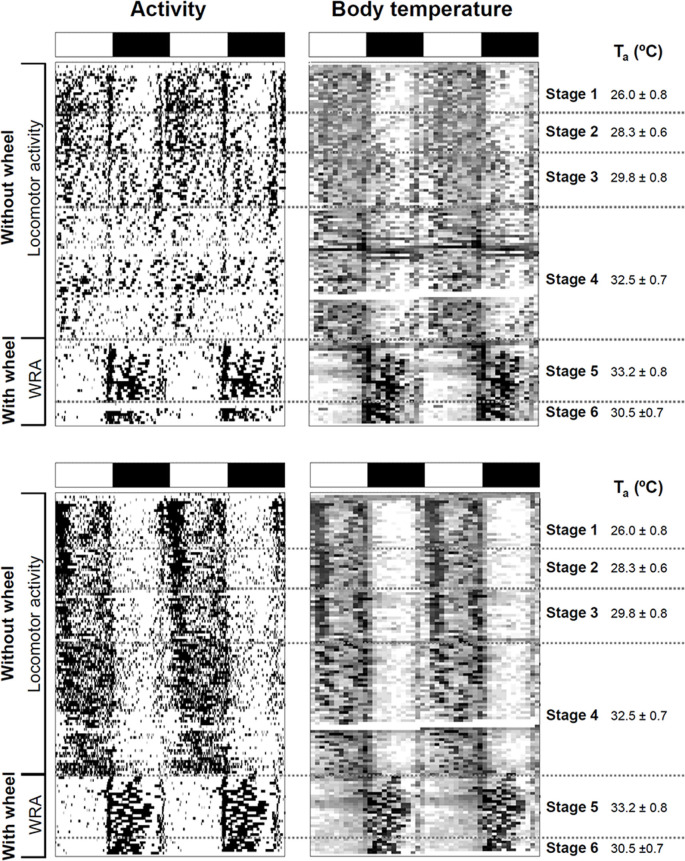
Impact of ambient temperature on behavioral and physiological rhythms in degus. Activity and body temperature actograms (left and right panels, respectively) from two degus kept under a 12:12 light-dark (LD) cycle without and with wheels. Experimental stages and ambient temperatures (T_a_: mean ± SD) are indicated on the right side of the actograms. LD cycle is represented at the top of each graph by white and black bars, respectively. Notice that in the activity actograms, locomotor activity data are plotted for stages 1–4, and wheel-running activity (WRA) for stages 5 and 6.

**Figure 2:**
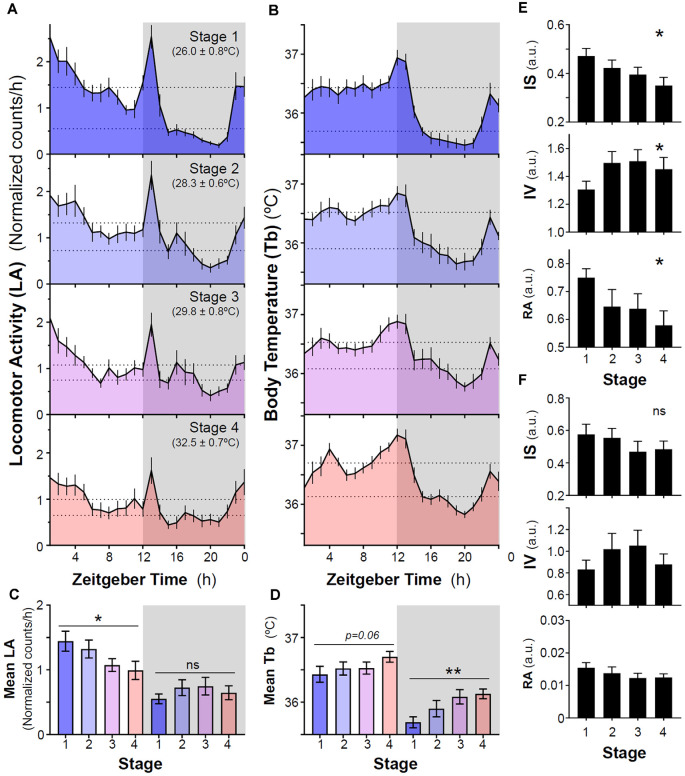
Impact of increasing ambient temperature on locomotor activity and body temperature rhythms in degus. **(A)** Locomotor activity and **(B)** body temperature (Tb) mean waveforms from degus kept without wheels under different ambient temperatures (Stage 1–4, T_a_: mean ± SD). Dashed lines indicate mean values during day and night. **(C)** Mean locomotor activity and **(D)** Tb during light and dark phases across different experimental stages. Non-parametric analysis of activity **(E)** and Tb **(F)** rhythms: inter-daily stability (IS), intra-daily variability (IV), and relative amplitude (RA) from stages 1–4. Data are plotted as mean ± SEM (*n* = 14 and 10 for LA and Tb, respectively). *p < 0.05, **p < 0.01, RM-ANOVA. Gray areas indicate the period of darkness. Zeitgeber Time 0 (ZT0) corresponds to timing of lights on. ns, not significant.

Our results show that a sequential increase in ambient temperature from 26°C to 32°C did not induce nocturnalism in naïve degus that never had previously wheel running experience. In general, animals exhibited predominantly a diurnal pattern of locomotor activity and body temperature (Tb) with two crepuscular peaks around dusk and before lights-on (Figures 1, 2A,B). Overall, in stages without wheel access, locomotor activity and mean Tb were higher during the light phase than at night (Figures 2A,B). However, sequential increases in ambient temperature resulted in a reduction of activity levels during the light phase, whereas levels at night remained unaltered (*p* = 0.0322 and 0.2245, respectively, RM-ANOVA, Figures 2A–C). In addition, locomotor activity rhythm became less stable (lower inter-daily stability, IS), more fragmented (higher intra-daily variability, IV) and with a lower relative amplitude (RA) when increasing ambient temperature (*p* = 0.0104, 0.0166 and 0.0282 for IS, IV, and RA, respectively, RM-ANOVA, Figure 2E). By contrast, there was a trend for animals to increase their Tb following ramping of ambient temperature, although this only reached statistical significance for mean Tb at night (*p* = 0.0602 and *p* = 0.0011, RM-ANOVA for Tb during light and dark phases, respectively, Figure 2D). No significant differences were found for IS, IV, and RA in Tb rhythm over the entire period (Figure 2F).

**Figure 3:**
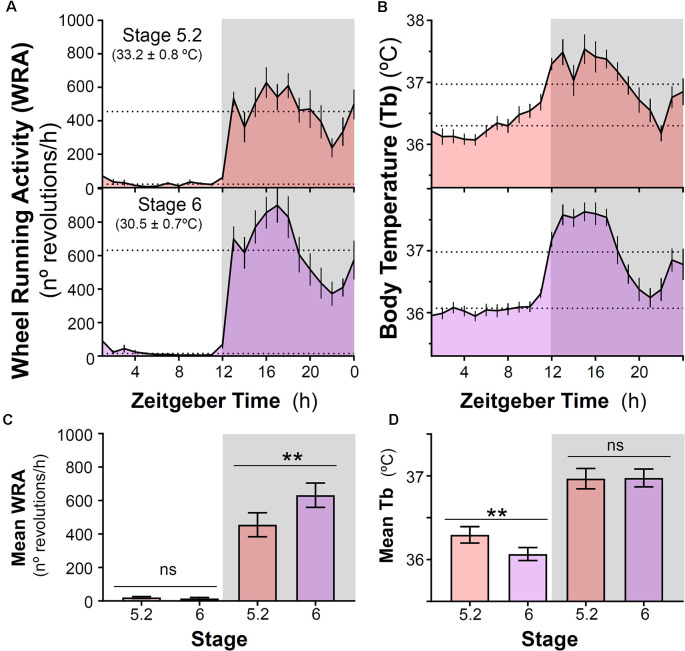
Impact of decreasing ambient temperature on wheel-running activity and body temperature rhythms in degus.** (A)** Wheel running activity (WRA) and **(B)** body temperature (Tb) mean waveforms from degus kept with wheels under two different ambient temperatures (Stage 5.2–6, T_a_: mean ± SD). Dashed lines indicate mean values during day and night. **(C)** Mean WRA and **(D)** Tb during light and night phases across the two stages. Data are plotted as mean ± SEM (*n* = 16 and 10 for WRA and Tb, respectively). **p ≤ 0.001, paired *t*-test following two-way ANOVA (*p* < 0.0125 was considered to be statistically significant). Gray areas indicate the period of darkness. Zeitgeber Time 0 (ZT0) corresponds to the time of lights on. Please, notice that at stage 6 (30.5 ± 0.7°C), animals remained nocturnal even though ambient temperature was lower than in stage 4 (prior to wheel availability, 32.5 ± 0.7°C). ns, not significant.

After introducing running wheels for the first time under high ambient temperature (~32–33°C), degus shifted their Tb rhythm and became nocturnal with higher Tb at night compared to daytime. In parallel with this nocturnal Tb pattern, animals confined their WRA to the dark phase (Figures 1, 3A,B) showing very low levels during the day (*p* < 0.001, the main effect of time of day; day vs night for Tb and WRA in stages 5.2 and 6, two-way ANOVA, Figures 3C,D). The analysis also revealed a significant time of day × stage interaction (*p* = 0.001 and *p* = 0.005 for WRA and Tb, respectively). Follow-up comparisons between stages for day or night showed that, when ambient temperature was reduced from ~33°C to 30°C at stage 6, animals increased their WRA levels at night, but showed no changes in Tb (Figures 3C,D, *p* = 0.0019 and *p* = 0.88 for WRA and Tb, respectively, paired *t*-test). However, during the daytime, a reduction in Tb occurred in parallel with the drop in ambient temperature, while WRA levels remained the same (Figures 3C,D, *p* = 0.001 and *p* = 0.3225 for Tb and WRA, respectively, paired *t*-test). Importantly, mean Tb values during the light phase in stages with wheel access (stage 5 and 6) are comparable to Tb at night-time in stages 3 and 4 (no wheels), being ambient temperature at stages 4 vs 5 (~32–33°C) and 3 vs 6 (~29–30°C) very similar (stage 4-mean night Tb = 36.13 ± 0.075°C vs stage 5.2-mean day Tb = 36.30 ± 0.098°C; stage 3-mean night Tb = 36.08 ± 0.111°C vs stage 6-mean day Tb = 36.07 ± 0.077°C; *p* > 0.05, paired *t-test*, for both comparisons, *n* = 10). This shift in the Tb pattern suggests that when animals get access to wheels, they not only run at night but overall become more active at night in terms of general activity. Interestingly, maximum Tb values during vigorous wheel-running activity were significantly higher than when animals were housed without wheels, even under similar ambient temperatures [Maximum Tb: stage 3 (without wheels) vs. stage 6 (with wheels): 37.14 ± 0.10 vs. 37.90 ± 0.14°C, *p* = 0.001, paired *t*-test].

**Figure 4:**
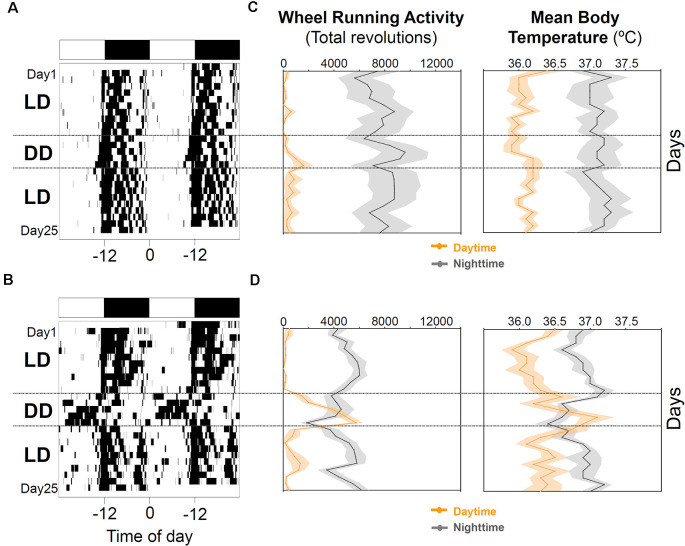
Nocturnal chronotype characterization. Wheel running activity actograms for representative entrained **(A)** and masked **(B)** nocturnal degus. Following 10 days under the 12:12 light-dark (LD) cycle (stage 6), animals were released into constant dark (DD) for 5 days and returned to the previous LD cycle for 10 more days. White and black bars at the top of each actogram represent the LD cycle. **(C,D)** The daily evolution of total activity (left panels) and mean body temperature (right panels) during daytime (orange line) and nighttime (gray line) for each group of nocturnal degus: **(C)** entrained (EN) and** (D)** masked (MN). Data are expressed as mean ± SEM (WRA, *n* = 7 and 9; Tb, *n* = 5 and 5, for EN and MN, respectively). Notice the increase in activity and body temperature during the subjective day in MN under DD conditions compared to EN.

**Figure 5:**
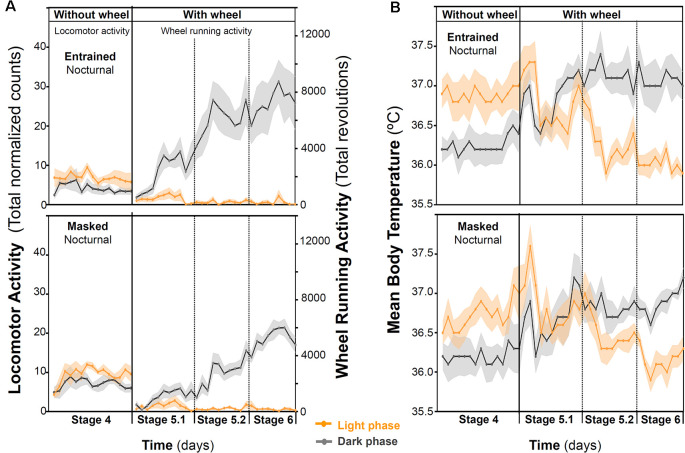
Behavioral and physiological changes in response to wheel-running availability in degus. The daily evolution of the total activity **(A)** and mean body temperature **(B)** during the day (orange line) and night (gray line) for each group of nocturnal degus: entrained (EN) and masked (MN). Data are expressed as mean ± SEM (WRA, *n* = 7 and 9; Tb, *n* = 5 and 5, for EN and MN, respectively). Notice that in the activity panel **(A)**, locomotor activity data are plotted for stage 4, and for stages 5 and 6, plotted data correspond to wheel-running activity.

When animals were released under DD, 7/16 animals (43.75%) started to free run from their former nocturnal phase in LD (entrained nocturnal; EN). By contrast, nine out of 16 degus (56.25%, masked nocturnal; MN) showed a phase advance from the first days under DD so that they mainly ran during their subjective day (Figure 4). The daily evolution of total activity during light and dark phases for EN and MN nocturnal degus is shown in Figure 5A. EN degus displayed a well-established nocturnal WRA pattern sooner than MN (Figure 5A). Indeed, in stage 5.2 (after 10 days with wheel), EN WRA rhythm was more robust (*p* = 0.007 for IV, unpaired *t*-test) and of higher amplitude (*p* = 0.042, unpaired *t*-test) compared to MN animals (Table 1). In the case of day-by-day evolution of mean Tb during light and dark phases (Figure 5B), EN also showed a well-established nocturnal Tb rhythm sooner than MN. However, differences in inter-daily stability, intra-daily variability, and relative amplitude between both nocturnal chronotypes were not significant (Table 1).

**Table 1:** Non-parametric analysis of activity and body temperature rhythms: inter-daily stability, intra-daily variability, and relative amplitude for entrained or masked nocturnal degus, at the different experimental stages with wheel access.

		Interdaily Stability (IS)		Intradaily Variability (IV)		Relative Amplitude (RA)	
		Entrained	Masked	Entrained	Masked	Entrained	Masked
Wheel	Stage 5.1	0.367 ± 0.042^A^	0.384 ± 0.052^A^	1.445 ± 0.061^A^	1.522 ± 0.104^A^	0.872 ± 0.072	0.893 ± 0.072
Running	Stage 5.2	0.555 ± 0.026^B^	0.465 ± 0.037^B^	1.102 ± 0.071^B^	1.432 ± 0.074^A^*	0.991 ± 0.006	0.951 ± 0.015*
Activity	Stage 6	0.543 ± 0.066^AB^	0.609 ± 0.033^C^	0.899 ± 0.148^B^	1.080 ± 0.118^B^	0.988 ± 0.011	0.975 ± 0.018
Body Temperature	Stage 5.1	0.379 ± 0.057^A^	0.356 ± 0.056	1.065 ± 0.193	0.945 ± 0.077	0.0119 ± 0.002	0.0101 ± 0.001
	Stage 5.2	0.522 ± 0.074^AB^	0.409 ± 0.051	1.077 ± 0.207	1.132 ± 0.077	0.0184 ± 0.003	0.0125 ± 0.003
	Stage 6	0.573 ± 0.053^B^	0.530 ± 0.062	0.918 ± 0.143	0.975 ± 0.150	0.0182 ± 0.003	0.0140 ± 0.002

*Values are expressed as mean ± SEM. Asterisks indicate significant differences between chronotypes (*p* < 0.05, unpaired *t*-test). Different letters indicate significant differences across stages for the same group (*p* < 0.05, RM-ANOVA, Tukey’s *post hoc* test)*.

**Figure 6:**
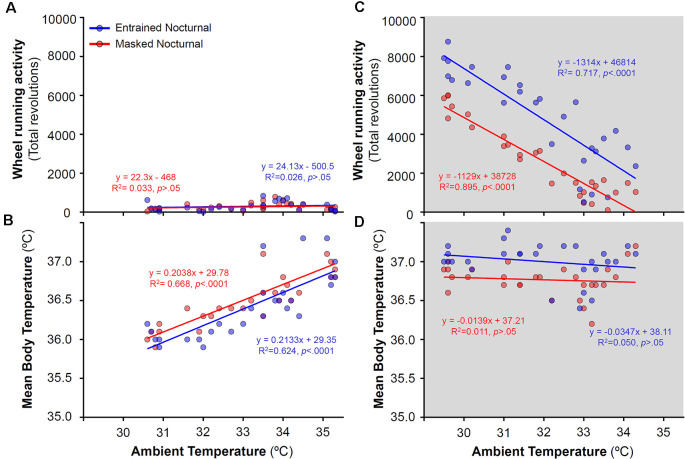
Relationship between daily wheel-running activity or body temperature and ambient temperature during the day **(A,B)** and night **(C,D)** in entrained (blue) and masked (red) nocturnal degus for each particular day across stages with wheel access (stages 5 and 6). Gray areas indicate darkness. Significant relationships identified by linear regression (*p* < 0.05).

**Figure 7:**
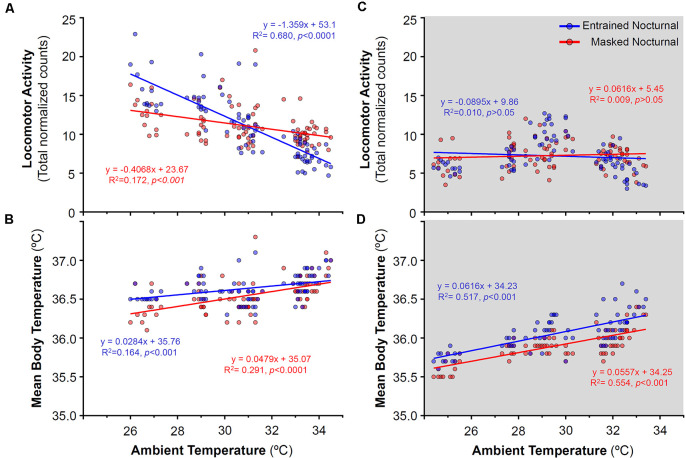
Relationship between daily locomotor activity or body temperature and ambient temperature during the day **(A,B)** and night **(C,D)** in entrained (blue) and masked (red) nocturnal degus for each particular day across stages without wheel access (stages 1–4). Gray areas indicate darkness. Significant relationships identified by linear regression (*p* < 0.05).

To analyze the relationship between activity levels and Tb with ambient temperature, daily mean Tb and total activity for both, EN and MN, degus were correlated with ambient temperatures for each particular day across the different stages. Correlations were performed separately for experimental stages with and without running wheels (Figures 6, 7, respectively). During wheel running access, a strong correlation was found between ambient temperature and WRA levels at night (*R*^2^ = 0.895 for MN and *R*^2^ = 0.717 for EN, Figure 6C) but not with Tb (Figure 6D). Indeed, both EN and MN ran more as the ambient temperature became cooler, with EN exhibiting overall higher WRA than MN (Figure 6C). During the light phase, mean Tb showed a reduction with decreasing ambient temperature (Figure 6B), but without a parallel decrease in WRA levels (Figure 6A).

During the stages without wheels, stages 1–4 (when degus were diurnal), no correlation was found between locomotor activity and ambient temperature at night (Figure 7C), that is, activity levels remained unchanged as the ambient temperature rose. By contrast, during the light phase, animals which would become EN when provided wheels, showed a strong reduction in locomotor activity with ramping ambient temperature (Figure 7A). During these stages without wheel access, mean Tb showed a moderate positive correlation with ambient temperature (Figures 7B–D).

## Discussion

The present study shows that naïve degus do not shift their diurnal activity and body temperature patterns to a nocturnal phase when they are subjected to sequential increases in the ambient temperature. However, when provided with wheels under high-temperature conditions, animals inverted their diurnal phase preference, and two different nocturnal chronotypes, entrained (EN) and masked (MN), emerged. The thermoregulatory response to wheel running during light and dark phases was significantly different in both chronotypes. To the best of our knowledge, this is the first comprehensive study addressing the behavioral thermoregulation in degus kept in the laboratory under different ambient temperatures that lie within their thermoneutral zone (26°C–32°C) and to investigate its relationship with wheel running availability.

Homoeothermic animals, such as the degus, present different coping mechanisms to keep their Tb within the physiological range. One of the most important strategies adopted to maintain thermal homeostasis involves adaptive behavioral responses (Fogo et al., 2019). *Octodon degus* is a caviomorph rodent from Central Chile, which has been described as diurnal with major episodes of activity at dawn and dusk (Fulk, 1976). Fulk (1976) suggested that degu is most active during daylight hours that offer less temperature stress. This view was supported by more recent field studies showing that degus adopt a unimodal day-active behavior during winter months, but shift their activity to a crepuscular pattern in the hot summer (Kenagy et al., 2002).

It is reported that degus’ body temperature, both in the field and in the lab, remains essentially between 36 and 38°C (Rosenmann, 1977; Refinetti, 1996; Kenagy et al., 2004), while degus’ thermoneutral zone ranges from 24 to 32°C. Due to their high water conservation and therefore low capability for evaporative cooling to successfully maintain this Tb range, degus avoid exposure to high ambient temperatures by retreating to a cooler environment (Rosenmann, 1977).

In our study, we subjected degus to different ambient temperatures ranging from 24°C to the upper limit of their thermoneutral zone (32°C). Our results show that activity levels in degus are reduced when increasing ambient temperature, particularly at 32°C. In the field, high environmental temperature causes the animals to avoid open areas and to seek shady places or retreat into their burrows to avoid thermal stress/ overheating (Lagos et al., 1995; Kenagy et al., 2004). Since these options are not available in lab conditions, our results show that the strategy adopted by degus to keep their Tb within the normal tolerance range when they are exposed to high temperatures was to reduce their daily activity and, therefore, reduce heat production. This behavioral strategy assures that degus are not pressed to their physiological temperature tolerance limits.

Together, lab and field studies identify the importance of thermoregulation as a key factor in the chronotype flexibility of the degus. Previous work has shown that degus can switch from diurnal to nocturnal activity in response to wheel running availability (Kas and Edgar, 1999; Ocampo-Garcés et al., 2005). In the present study, when wheels were provided under high ambient temperature, all animals showed a nocturnal WRA pattern. It is becoming apparent that wheel-running activity and general cage activity do not represent the same behavior (Novak et al., 2012). Although, with our methodology, we could not monitor simultaneously general activity and WRA, the shift in Tb rhythms to the nocturnal phase (with low values during daytime) after providing wheels argues in favor that degus become nocturnal in terms of both general activity and WRA.

Overall, our data support the previous prediction from Lee’s lab suggesting that the relative proportion of degu’s chronotypes (diurnal/nocturnal) may be dependent on ambient temperature with more crepuscular and nocturnal patterns in degus as room temperature rises (Lee, 2004; Hagenauer and Lee, 2008). The simultaneous presence of diurnal and nocturnal degu chronotypes in response to wheel running availability has been reported in animals kept at room temperatures between 21 and 24°C (Refinetti, 2006; Vivanco et al., 2009; Otalora et al., 2010). Thus, wheel running availability and ambient temperature may account for the different patterns and proportions of each chronotype reported by different labs.

It has been postulated that shifting activity to the nocturnal phase in response to wheel running could be one way of attaining high levels of activity while avoiding the danger of overheating. Vivanco et al. (2010c) showed that temperature cycles, with high values during the day and low values at night, induce nocturnal chronotypes in degus previously characterized as diurnal. However, in the present study degus selected to be active during the dark phase even when daily ambient temperature fluctuations were minimal, and thus no advantage to be active at night would be obtained. These data support the view proposed by Kas and Edgar (1999), that an innate “protective” pre-program mechanism, that implies associating dark phase with lower ambient temperature, changes the timing of the behavioral activity in this animal species to reduce the potential risk of hyperthermia.

Based on the phase relationship of the activity rhythm under LD and DD, EN and MN animals can be differentiated. Thus, steady entrainment and negative masking effects by light would explain the gradient of circadian chronotypes in degus, from diurnal to nocturnal (Vivanco et al., 2009, 2010b,d; Otalora et al., 2013). In our study, we found a very similar proportion of animals exhibiting a masked or entrained nocturnal pattern. These percentages meet with those previously reported by Vivanco et al. (2009) in animals kept at 23°C. Since our degus were maintained at 32°C, it seemed that high ambient temperature by itself does not produce the differentiation to any nocturnal chronotype in particular.

The present study reports that EN degus exhibited a well-established WRA pattern sooner than the MN animals. In addition, EN runs more than MN without involving great changes in Tb. These results indicate that the thermoregulatory response to wheel running availability, at high room temperature, may differ among these chronotypes. However, the mechanisms underlying these differences remain unknown. Vivanco et al. (2009) also demonstrated that there were some differences in the thermal responses to intense wheel exercise between nocturnal and diurnal degus and that those differences seem to be induced after wheel running availability.

In conclusion, diurnal degus did not shift their activity and Tb pattern to the nocturnal phase in response to a gradual rise in ambient temperatures but all animals became nocturnal when, in addition to a high ambient temperature close to their upper thermal tolerance limit, running wheels were introduced. This nocturnal pattern was achieved through two different mechanisms: some degus, EN, show a pacemaker entrained to the scotophase while others, MN, exhibit a pacemaker entrained to the photophase, but with a strong negative masking effect by light.

Nocturnal entrainment appears to allow degus to perform more vigorous physical activity than that displayed by MN degus, with the same thermal increase. Although it is clear that the appearance of nocturnal chronotype could be an adaptive response to thermoregulatory constraints, the biological significance of the existence of two mechanisms involved in the nocturnalism of the degus remains unclear. It can be thought that the degu is a diurnal species that evolved from a nocturnal pattern but still conserving a highly flexible temporal niche preference that could be advantageous in nature.

## Data Availability Statement

The datasets generated and analyzed for this study are available from the corresponding author on reasonable request.

## Ethics Statement

All animal work was performed in accordance with the guidelines issued by Spanish Ministry of Agriculture, Fishing and Feeding (Royal Decree 1201/2005 of October 21, 2005) and was approved by the Bioethical Committee of the University of Murcia (Spain).

## Author Contributions

BB-O, MR, and JM designed the study, wrote the manuscript, and acquired funding. BB-O performed the experiments. All authors contributed to the article and approved the submitted version.

## Conflict of Interest

The authors declare that the research was conducted in the absence of any commercial or financial relationships that could be construed as a potential conflict of interest.
